# The impact of 3-dimensional models and surgical navigation for open liver surgery

**DOI:** 10.1007/s11548-025-03455-5

**Published:** 2025-07-01

**Authors:** Karin A. Olthof, Matteo Fusaglia, Anne G. den Hartog, Niels F. M. Kok, Theo J. M. Ruers, Koert F. D. Kuhlmann

**Affiliations:** 1 Department of Surgical Oncology, The Netherlands Cancer Institute – Antoni van Leeuwenhoek, Plesmanlaan 121, 1066CX Amsterdam, The Netherlands; 2 Faculty of Science and Technology (TNW), Nanobiophysics Group (NBP), University of Twente, Drienerlolaan 5, 7522 NB Enschede, The Netherlands

**Keywords:** Liver surgery, Surgical navigation, Computer-assisted surgery, 3D models, Intraoperative decision support

## Abstract

**Purpose:**

Understanding patient-specific liver anatomy is crucial for patient safety and achieving complete treatment of all tumors during surgery. This study evaluates the impact of the use of patient-specific 3D liver models and surgical navigation on procedural complexity in open liver surgery.

**Methods:**

Patients with colorectal liver metastases scheduled for open liver surgery were included between June 2022 and October 2023 at the Netherlands Cancer Institute. Patient-specific 3D liver models could be used upon request during the surgical procedure. Subsequently, surgeons could request additional surgical navigation by landmark registration using an electromagnetically tracked ultrasound transducer. Postoperatively, surgeons assessed the impact of the use of the model and navigation on procedural complexity on a scale from 1 to 10.

**Results:**

35 patients were included in this study, with a median number of 8 (ranging from 3 to 25) tumors. 3D models were utilized in all procedures. Additional navigation was requested in 21/35 of patients to improve intraoperative planning and tumor localization. The mean procedural complexity score with navigation was 4.3 (95% CI [3.7, 5.0]), compared to 7.8 (95% CI [6.6, 9.0]) with the 3D model alone. Both visualization methods improved lesion localization and provided better anatomical insight.

**Conclusion:**

3D models and surgical navigation significantly reduce the complexity of open liver surgery, especially in patients with bilobar disease. These tools enhance intraoperative decision-making and may lead to better surgical outcomes. The stepwise implementation of the visualization techniques in this study underscores the added benefit of surgical navigation beyond 3D modeling alone, supporting its potential for broader clinical implementation.

**Supplementary Information:**

The online version contains supplementary material available at 10.1007/s11548-025-03455-5.

## Introduction

During liver surgery, complete thermal ablation or radical surgical resection of all tumors is key to achieve optimal oncologic outcome. It is imperative to try to prevent vascular or biliary injury. Accurate localization of tumors and their relation to vital anatomical structures is crucial to ensure adequate local treatment. Intraoperative ultrasound (US) is the tool surgeons frequently use to assess intrahepatic anatomy and to localize deep-seated tumors. Identification of liver lesions using US can however be challenging in patients with widespread metastases, steatotic or cirrhotic liver parenchyma or in small or vanished lesions after good response to systemic therapy [1–3]. When lesions cannot be identified intraoperatively, up to half of the surgeons estimate the location of the lesion for local treatment, based on diagnostic imaging and surgical experience, while the other half opts for observation and no treatment [4]. This depends on location of the tumor and local preferences. When left untreated, regrowth occurs in a third of the initially vanished metastases [5].

3D models have become widely accepted for improved understanding of spatial relationship between tumors and critical surrounding anatomy. They facilitate preoperative planning and have shown to reduce operation time, extent of the surgical resection, and blood loss [6–9]. In addition, these models can be merged with intraoperative imaging such as ultrasound, a technique known as image-guided surgery or surgical navigation. The patient’s liver position and orientation is then spatially mapped to the preoperative images using a process called registration, to provide real-time, 3D guidance.

Even though the use of 3D liver models is implemented in many centers, the adoption of surgical navigation is still limited because only a few centers possess the necessary technology and expertise. Consequently, there is limited scientific evidence supporting the clinical benefits of the technique. To evaluate whether widespread use would be of value, we assessed the effectiveness of surgical navigation in open liver surgery and aimed to determine if this technology could reduce complexity of the procedure.

For a description and demonstration of the techniques employed in this study, the reader is referred to [10, 11].

## Methods

### Patient population

This study was conducted in accordance with ethical principles and received approval from the Institutional Review Board (IRBd25-001). This single-center prospective study included consecutive patients of 18 years or older with colorectal liver metastases at the Netherlands Cancer Institute from June 2022 to October 2023. All patients were scheduled for open liver surgery, undergoing resection and/or ablation of more than three tumors. The Netherlands Cancer Institute is a specialized cancer hospital where complex surgical procedures of patients with a high number of metastases are routinely performed. The use of 3D models and surgical navigation is standard of care at our institute, and therefore, no specific informed consent was required for its use. Patients scheduled for an anatomical hemihepatectomy were excluded. In addition, patients with pacemakers were excluded due to the potential interference of the electromagnetic tracking system.

### 3D segmentation

MRI or CT scans of each patient were used to segment the liver parenchyma, tumors, vasculature, biliary tree, and benign lesions such as hemangiomas or cysts. In addition, postoperative areas (resected areas or ablation zones) from previous procedures were included in the models. For MRI, delineation was made using the hepatobiliary phase with the assistance of an automatic segmentation algorithm [12, 13] and subsequently refined manually with the Segment Editor module in 3D Slicer [14]. For CT scans, 3D models were produced by automatically segmenting the liver and vasculature and semi-automatically segmenting tumors using the Liver Segmentation software module from IntelliSpace Portal® (Philips Healthcare, the Netherlands).

### Surgical procedure

**Fig. 1:**
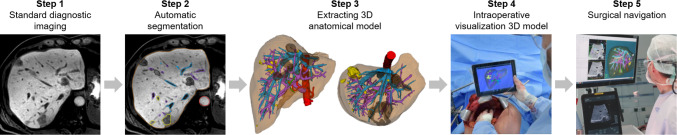
Study steps: workflow of creating and visualizing 3D models and surgical navigation. 3D models include parenchyma (brown), portal vein (purple), hepatic vein (blue), hepatic artery (red), bile ducts (green), postoperative areas from previous procedures (black) and tumors (yellow)

Intraoperatively, a curved array US transducer (type I14C5T, BK Medical, Denmark) was used to identify liver lesions. Contrast-enhancement was not used. A calibrated adapter containing an EM sensor was attached onto the ultrasound transducer, which was subsequently covered with a sterile drape. Surgeons were not provided with the 3D model unless requested. When requested, the 3D model was provided on a touchscreen tablet enclosed in a sterile cover or on a navigation display above the surgical field. After surgeons used the 3D model, surgical navigation was only initiated upon request from the surgeon (Fig. 1).

### Navigation workflow

The Aurora V2 electromagnetic planar field generator (Northern Digital Inc., Waterloo, Ontario, Canada) was positioned close to the surgical field. Continuous liver tracking was enabled by attaching a sensor to the liver in proximity of the targeted lesion using glue. This aimed to compensate movement of the liver due to breathing or surgical manipulation. During the registration process, the position of clearly recognizable landmarks, such as vessel bifurcations, were recorded in the US image as well as in the preoperative model. Navigation information was visualized as a virtual scene depicting the liver model and tracked surgical instruments, and the US video stream augmented with a projection of the registered 3D model. The surgical navigation workflow is provided in Video 1.

### Outcomes

The procedures were performed by four hepatobiliary surgeons. For each procedure, one of the surgeons was postoperatively asked to evaluate the 3D visualization techniques and report about the added value of their use during open liver surgery. Surgeons were asked to grade the complexity of the procedure on a 10-point scale (with 1 representing the simplest and 10 the most complex procedures) with and without the aid of 3D models and with or without surgical navigation, and to specify how these techniques influenced the complexity of the procedure.

## Results

**Fig. 2:**
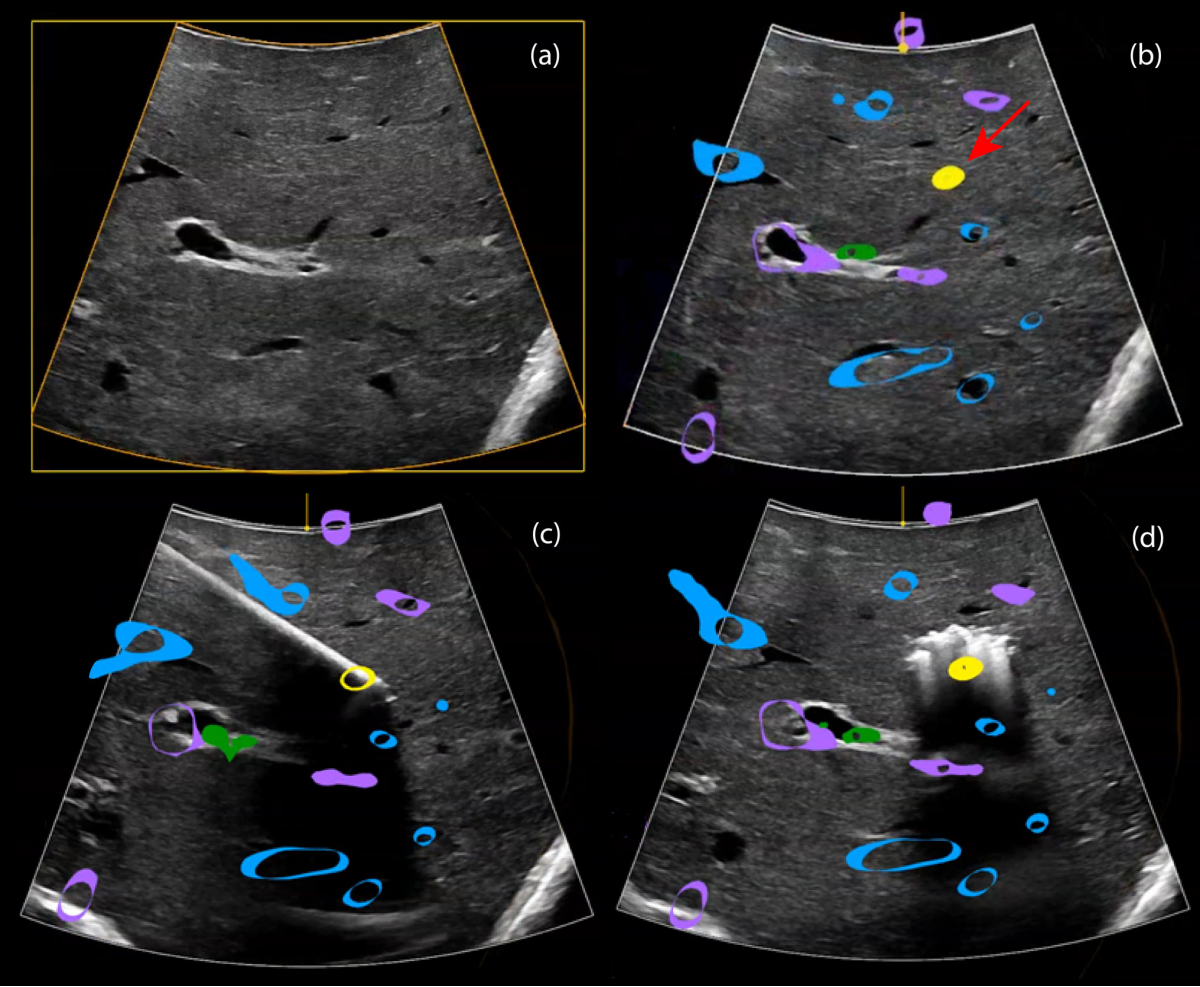
Steps of localization and treatment of a vanished lesion on intraoperative ultrasound using surgical navigation, with **a** standard ultrasound imaging, **b** localization of vanished lesion by projection of the patient-specific 3D model on the live ultrasound image, **c** ablation antenna inserted in vanished lesion based on surgical navigation, and **d** gas formation after microwave ablation

35 patients were included in this study, with a median number of 8 (ranging from 3 to 25) tumors. The surgeons requested the liver model in all procedures. Two patients were excluded from further evaluation due to substantial disease progression observed at laparotomy, causing a discrepancy between the current disease status and the 3D model. Navigation was requested in 20 of 33 patients to confirm the location of lesions or to identify those that were not found using intraoperative US and 3D model (Video 2). After localization, lesions were either resected or ablated based on the fusion of the 3D and the intraoperative US (Fig. 2). Patient characteristics are shown in Table 1, distinguishing between those treated only with the aid of the 3D model and those treated using surgical navigation. There were no statistically significant differences between the two groups.

**Table 1:** Patient characteristics

Surgical procedures	Model Only (n = 13)	Model and navigation (n = 20)	*P*
Sex, M / F	11/2	14 / 6	0.34
Age (years), median [range]	61 [36–75]	56 [39–76]	0.64
Preoperative treatment			0.80
None	1 (7.7)	1 (5.0)	
Systemic chemotherapy	7 (5.4)	9 (45.0)	
Hepatic arterial infusion pump (HAIP) combined with systemic chemotherapy	5 (38.5)	10 (50.0)	
Number of tumors per patient, median [range]	8 [3–25]	8 [3–18]	0.97
Number of resections	4 [0–9]	2 [1–11]	0.38
Number of ablations	4 [0–16]	6 [2–12]	0.63

**Fig. 3:**
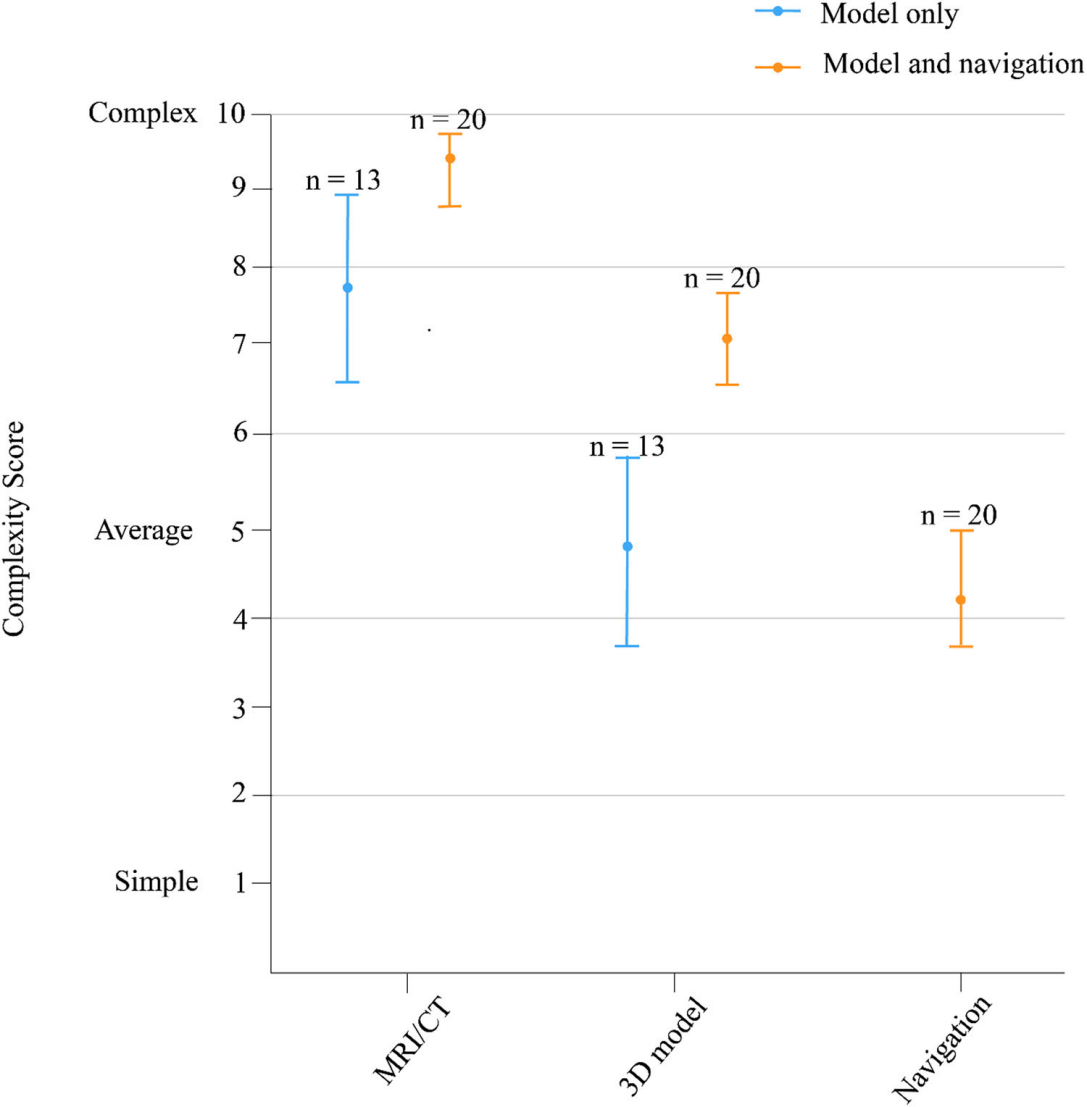
Survey findings on the impact of 3D modeling and surgical navigation on the procedural complexity of open liver surgery, showing the mean and 95% confidence interval

The use of the two 3D visualization techniques facilitated complex liver procedures (Fig. 3). In the group where no navigation was requested (the “Model only” group), the use of the 3D model led to a significant decrease in the complexity of the procedure as scored by surgeons. Nevertheless, in most patients, the 3D model alone was not sufficient for surgical guidance. The procedures were still complex even when aided by the 3D model (complexity score = 7.1, 95% CI [6.5, 7.7] and navigation was additionally requested (the “Model and navigation” group).

Procedures in the “Model and navigation” group were on average more complex than those in the “Model only” group (Model and navigation mean complexity score = 9.4, 95% CI [8.9, 9.9] vs. Model only mean complexity score = 7.8, 95% CI [6.6, 9.0]). However, both groups converged to similar complexity scores when navigation was used (Model only mean complexity score = 4.6, 95% CI [3.6, 5.6] vs. Model and navigation mean complexity score = 4.3, 95% CI [3.7, 5.0]). In addition, surgeons were asked to specify how these techniques influenced the surgical complexity. Decision support and tumor localization were the primary reason for both 3D modeling and surgical navigation. This was particularly of use in patients with small and isoechoic lesions and in steatotic livers, in which differentiation tumor from healthy parenchyma can be very challenging. The use of 3D models additionally improved 3D insight of the tumor to surrounding anatomical structures and facilitated tracking of treated lesions in procedures involving a high number of lesions.

## Discussion

Although the pre- and intraoperative use of virtual models is widely accepted, integration of surgical navigation during liver surgery remains limited. The stepwise implementation of 3D models followed by surgical navigation in this study allowed for comparison of the two visualization methods. Aligning with literature, this study underscores the benefit of 3D models in liver surgery. Hepatobiliary surgeons requested the use of 3D models in all procedures to improve insight in critical structures and tumor locations. In addition, the demand and importance of surgical navigation in liver procedures is demonstrated, especially in patients who received preoperative systemic treatment. Surgeons requested surgical navigation in 20 out of 33 of complex liver procedures and indicated that these procedures became easier using the technique.

In literature, several examples of surgical navigation for treatment of liver lesions have been reported, especially in patients with vanishing lesions [15–18]. Evaluation of navigation systems is therefore often performed based on localization efficacy or local recurrence rate. We previously demonstrated efficacy of localization and subsequent ablation in 33 out of 34 lesions not found using ultrasound alone [11]. After one year follow-up no difference was observed in local recurrence rates of visible lesions and invisible lesions treated based on surgical navigation. Similarly, Kingham et al. [15] evaluated the Explorer (Analogic Corporation, Peabody, MA) image guidance system for localizing occult lesions on ultrasound. Out of 22 vanishing lesions, 15 lesions were localized using surgical navigation.

Evaluating rapidly evolving technologies is challenging due to the continuous process of improvements. While assessing effectiveness based on clinical outcomes and cost-effectiveness is important, it is also important to demonstrate clinical benefits that these technologies provide according to surgeons, such as facilitating procedures and enhancing decision-making. Usability questionnaires serve as a valuable tool for validating new technologies, but are not used frequently. For example, Groen et al. [18] showed good usability of a navigation system during resection of locally recurrent rectal cancer with an average standardized usability score of 71 ± 12 (range 0–100). To the best of our knowledge, no other studies have directly compared the use of 3D models alone with surgical navigation in liver surgery.

This study had several limitations. Most important limitation is that only the perceived surgical complexity was measured, while its relation to relevant clinical or oncological outcomes were not taken into account. Potential bias occurred because of the included patients, and the surgeons performing the procedures. At our institute, specialized cancer care is provided, and complex surgical procedures are routinely performed. The median number of metastases was high which contributed to the perceived average high complexity level without the use of 3D models and surgical navigation. Due to high complexity, surgeons were more inclined to request additional guidance. The hepatobiliary surgeons involved had significant experience with 3D models and surgical navigation. Experience most likely increased the likelihood of their utilization. Transferring this concept to other hospitals and measuring the effect and additional benefit without previous expertise is key. Moreover, the additional value in patients with perceived lower complexity liver surgery should be studied. This should incorporate short and long-term outcomes which reflect potential benefit to the patient. It is questionable whether randomization would be feasible once these technologies are available, although data could be aggregated in studies with a step wedge design.

In conclusion, this study demonstrated that 3D models and the additional integration of surgical navigation facilitated liver surgery in a significant subset of patients, particularly those with a high number of bilobar spread lesions and preoperative systemic treatment. Moving forward, close collaboration between clinicians and technicians is essential to further refine and enhance their integration into routine clinical practice.

## Supplementary Information

Below is the link to the electronic supplementary material.

Supplementary file1 (MP4 64376 KB)

Supplementary file2 (MP4 99891 KB)

## Data Availability

The datasets generated during and/or analyzed during the current study are not publicly available.
